# Influence of Lanthanum-Doped Tungsten Wire Drawing Process on Microstructure and Properties

**DOI:** 10.3390/ma15144979

**Published:** 2022-07-18

**Authors:** Luyan Wang, Yueguang Yu, Peng Zhang, Jiancan Yang

**Affiliations:** 1 School of Materials Science and Engineering, University of Science and Technology Beijing, Beijing 100083, China; b20150162@xs.ustb.edu.cn; 2 BGRIMM Technology Group, Beijing 100160, China; 3 China Iron & Steel Research Institute Group, Beijing 100081, China; 4 College of Materials Science and Engineering, Beijing University of Technology, Beijing 100124, China; naes@bjut.edu.cn (P.Z.); yjcan@bjut.edu.cn (J.Y.)

**Keywords:** lanthanum-doped tungsten wire, processing, microstructure, mechanical behavior

## Abstract

A reasonable preparation processing of Lanthanum-doped tungsten wire plays a decisive role in the final properties of the wire. This paper gives the optimum drawing process parameters of lanthanum-doped tungsten wire with φ1.00 mm–φ0.50 mm and explains the phenomenon of coarsening of fiber-like grains in the preparation processing of tungsten wire. The final optimum process parameters of lanthanum-doped tungsten wire are given: the temperature is 950 °C (the first pass temperature is 950 °C, and the temperature decreases by about 20 °C for each pass), the compression ratio is 15%, mold temperature is 550 °C, because of the limitation of equipment conditions, the wire drawing speed is fixed at 0.19 m/s. It is found that the fiber-like grains of the tungsten wire coarsen when the temperature is too high, and it is prone to breakage when the temperature is too low during the drawing process. When the compression ratio is too high (for example, 22%), there is a negative impact on the surface quality and the straightness of the tungsten wire. When the compression ratio is too low, the processing die time is increased, and the production cost is increased.

## 1. Introduction

When it was first invented, the magnetron was mainly used in radar and made important contributions to military development. With its expansion in the civilian field, in terms of output and output value, the application of magnetrons in the field of household microwave ovens ranks first. The world’s annual output of microwave magnetrons is about 100 million [1,2]. As a commonly used household appliance, microwave ovens still have a good market prospect in the future. The magnetron is the core component of the microwave oven, and the cathode is the core component of the magnetron. The main problem of the cathode is the process and the material composition. For the pressure processing of materials, researchers mainly focus on the effect of compression ratio and temperature [3]. According to one investigation, the main cathode material of the magnetron is still the thorium-doped tungsten cathode [4]. It is well-known that thorium is a radioactive element, especially in the process of its usage as a cathode, as it releases radioactivity continuously [5]. How to eliminate radioactive contamination and improve service life and stability are the two main problems faced by microwave magnetrons. In recent years, researchers have been actively looking for solutions. Sun Lingbin, Zhang Wenliang, and others studied thoriated tungsten carbide cathodes; they found that layered W_2_C and bulk WC structures have a huge difference in the quality of thoriated tungsten cathodes. By optimizing the carbonization process, the consistency of cathode carbonization was further improved. Thereby, the working stability of the magnetron is improved [6,7,8]. However, this technology does not solve the problem of radioactive contamination.

At present, the mainstream material that can replace the thorium-doped tungsten is rare-earth-doped tungsten alloy [5]. For example, lanthanum-doped tungsten can effectively improve the electron emission performance and high temperature performance of the material [9]. However, the incorporation of rare earth oxides hinders grain boundary migration and dislocation motion [10,11,12], which results in the lower processability of rare-earth-doped tungsten than pure tungsten and thorium-doped tungsten [13]. Additionally, in recent years of research, the technology of processing rare-earth-doped tungsten wire over φ1.00 mm has been well-developed. However, for large and fine wire, it is very difficult to process. Some scholars have conducted research on the forging process of large-scale tungsten–lanthanum rods [14]; the further enhanced processing of the tungsten wire smaller than φ1.00 mm has always met certain problems. As the cathode diameter of the microwave magnetron is about 0.50 mm, this paper would mainly study the preparation of φ1.00 mm–φ0.50 mm lanthanum-doped tungsten wire.

In this paper, the problems in the preparation process of lanthanum-doped tungsten wire in φ1.00 mm–φ0.50 mm are deeply explored and analyzed; this would provide theoretical support for the deep processing of multiple compound rare-earth-doped tungsten wire.

## 2. Materials and Experimental Methods

The materials investigated in this paper are lanthanum-doped tungsten wires. The raw materials are ammonium paratungstate and lanthanum nitrate.

Tungsten wire production process: doping–reduction–pressing–sintering–rotary forging–chain drawing (φ3.00 mm–φ1.00 mm)–large turntable wire drawing (φ1.00 mm–φ0.50 mm), and finally, a tungsten wire with a diameter of 0.5 mm is prepared. Since the earliest research on rare earth tungsten was based on the demand for tungsten electrodes of argon arc welding, such electrodes’ diameters are generally more than 1 mm, such as 1.6 mm, 2.4 mm, etc. As the products have achieved large-scale applications, which technology has matured, this paper mainly focuses on the analysis of the technics for the preparation of tungsten wire with φ1.00 mm–φ0.50 mm on a large turntable wire drawing machine.

A batch of lanthanum-doped tungsten wires with φ1.00 mm was prepared by using the same batch of materials and the same preparation process at first. Then, the large turntable wire drawing machine was used to prepare tungsten wires of φ0.50 mm under various processing conditions.

Tungsten wires with diameters of 0.50 mm were prepared by four different processes. The four process parameters are shown in Table 1.

Firstly, the properties of the tungsten wires prepared by different processes were tested for microhardness and straightness. The microhardness was measured with Vickers hardness. The load and the residence time used in the experiment were 200 g and 15 s, respectively. At the same time, 5 points were collected in the same spot, and then the average value was used as the result. The straightness is normally measured by hanging up a 1000 mm tungsten wire and measures its vertical distance. The greater the vertical distance, the better the straightness [15]. In this paper, a φ0.5 mm tungsten wire with a length of 1000 mm is used.

In order to study the relationship between properties and microstructure, the microstructure of the tungsten wire was observed by electron backscattered diffraction (EBSD). The samples used for further analyses were prepared by several steps. Firstly, the lanthanum-doped tungsten wire from different die times were cut to 0.80 mm using a wire-cutting method. Secondly, all the wire pieces were embedded in hard resin and then followed by mechanical polishing and ion thinning to smooth the surfaces. For all the wires, samples from different die times were prepared in two principal viewing directions: longitudinal section (plane parallel to the drawing axis) and cross section (plane perpendicular to the drawing axis).

The microstructure of the wire was studied by electron backscatter diffraction and field emission scanning electron microscopy (QUANTA FEG650) (American FEI, Hillsboro, OR, USA) using an accelerating voltage of 20 kV. In order to avoid data inaccuracy in one single area, information was collected from different regions, and all information from each area showed a similar microstructure.

## 3. Experimental Result

### 3.1. Mechanical Behavior

#### 3.1.1. Microhardness Measurements

**Figure 1:**
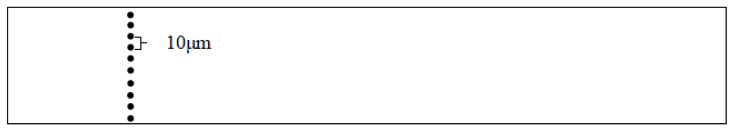
Schematic diagram of hardness test.

**Figure 2:**
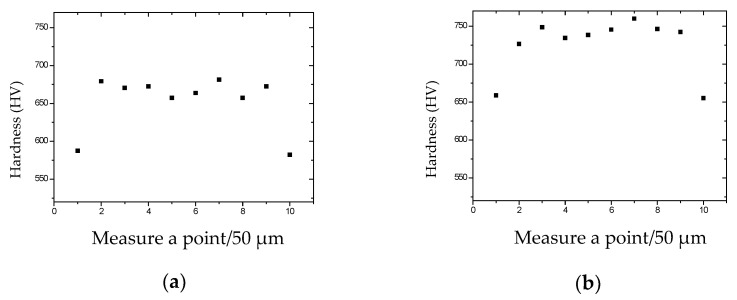

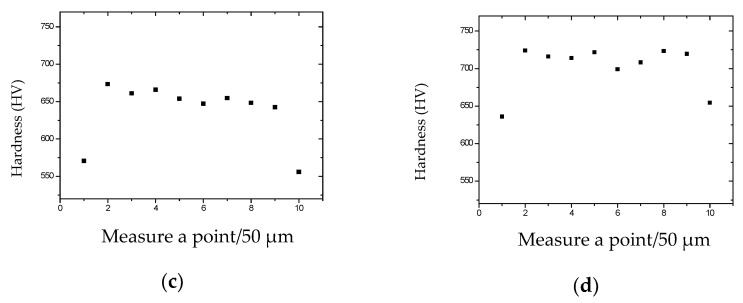
Microhardness data (**a**) φ0.50 mm tungsten wire prepared by Process 1, (**b**) φ0.50 mm tungsten wire prepared by Process 2, (**c**) φ0.50 mm tungsten wire prepared by Process 3, (**d**) φ0.50 mm tungsten wire prepared by Process 4.

The microhardness for the longitudinal section of the tungsten wires prepared by different processes was measured (measure a point per 50 μm, as shown in Figure 1), and the results are in Figure 2.

It can be seen that the microhardness of Process 1 and Process 3 is lower, and the microhardness of Process 2 and Process 4 is higher. At the same time, the microhardness of the edge area of the tungsten wire is significantly lower than in the intermediate area.

#### 3.1.2. Straightness Test

Generally, the magnetron cathode needs to be used after winding the wire. If the straightness of the tungsten wire is more than 40%, the straightness of the tungsten wires is considered to be good. If the straightness of the tungsten wires is low and the stress is high, it will distort and deform the cathode of the magnetron, and this will affect the performance. The straightness tests were carried out with the tungsten wires prepared under different processes. Table 2 shows the test results.

### 3.2. Microstructure Analysis

**Figure 3:**
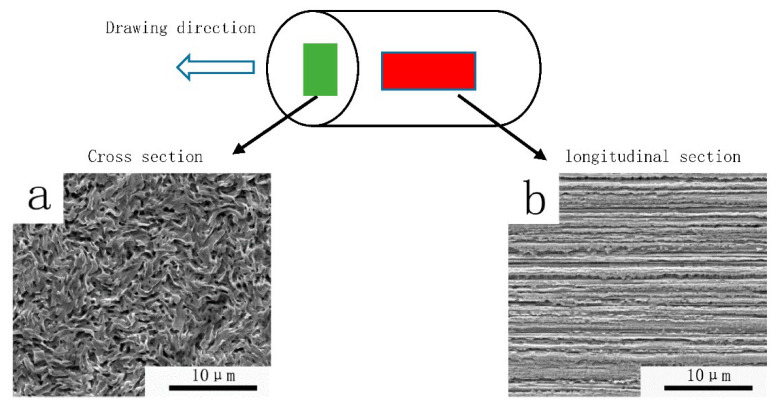
Scanning electron micrographs. (**a**) Cross section microstructure of wire. (**b**) Longitudinal section microstructure of wire.

As can be seen from Figure 3, the grains of the tungsten wire in the longitudinal section are long fiber-like grains, and grains in the cross section are curled instead of equiaxed. This indicates that during the preparation of the tungsten wires, the grains are elongated along the deformation direction, and the elongated intertwined grains composed of this special microstructure are gradually formed. The same experimental results are also repeated in E.S. Meieran and A. Barna’s papers [16,17].

**Figure 4:**
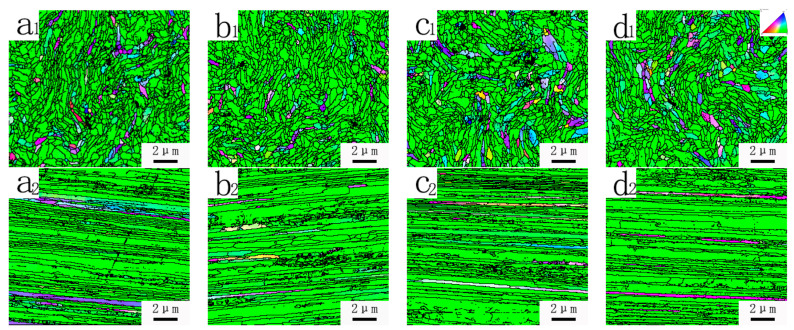
EBSD orientation maps (**a_1_**,**a_2_**) φ0.90 mm tungsten wire prepared by Process 1; (**b_1_**,**b_2_**) φ0.92 mm tungsten wire prepared by Process 2; (**c_1_**,**c_2_**) φ0.92 mm tungsten wire prepared by Process 3; (**d_1_**,**d_2_**) φ0.90 mm tungsten wire prepared by Process 4.

From the EBSD orientation map (Figure 4), it can be seen that the cross-sectional grain size of the tungsten wires prepared in the four different processes did not change significantly after the first mold. In this case, the displayed fiber width difference of the longitudinal section may be due to the different grain orientation. Therefore, only the cross section would be studied in the following contents.

Figure 4 shows that there is no significant difference for the microstructure of the lanthanum-doped tungsten wires prepared by chain drawing. At the same time, as a typical BCC metal structure, tungsten alloy produces a typical <110> texture under a large deformation [18].

**Figure 5:**
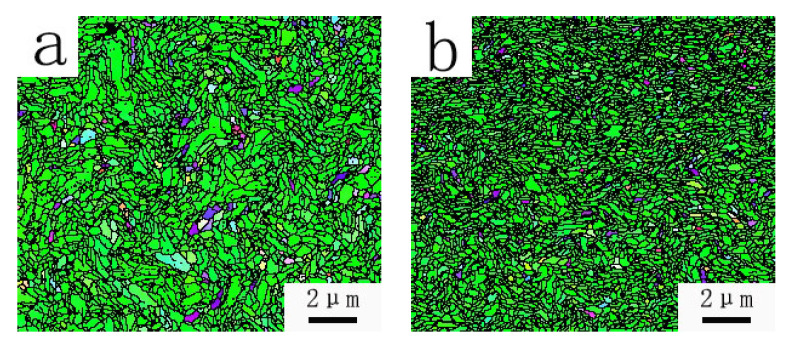

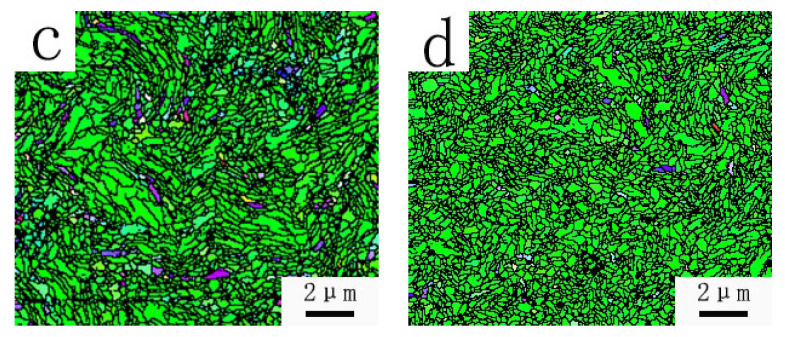
EBSD orientation maps. (**a**) φ0.50 mm tungsten wire prepared by Process 1, (**b**) φ0.50 mm tungsten wire prepared by Process 2, (**c**) φ0.50 mm tungsten wire prepared by Process 3, (**d**) φ0.50 mm tungsten wire prepared by Process 4.

As is shown in Figure 5, when the diameter of the tungsten wire is 0.5 mm, the size of the grains has a significant change. Using different wire drawing processes, tungsten wires with different grains size were obtained. The average sizes of the grains shown in Figure 5a–d are σ = 0.1684 μm, σ = 0.0982 μm, σ = 0.1361 μm, σ = 0.1024 μm. It can be seen that the sizes of grains prepared by Process 2 and Process 4 are much finer than those by Process 1 and Process 3.

It is noted in Shi Deke’s book [19] that the tensile strength and toughness for the structure of cold-deformed metal is high, and the shear strength is low, along with the direction of the fiber-like structure (longitudinal section), and this case is opposite when it is perpendicular to the fiber direction (cross section). In addition, the finer the fiber-like grains in the tungsten wire, the smaller the probability of defects, such as voids inside, and the higher the tensile stress that the whole wire can withstand. Therefore, we believe that tungsten filaments with finer fiber-like grains have better performance.

### 3.3. Surface Quality Inspection

**Figure 6:**
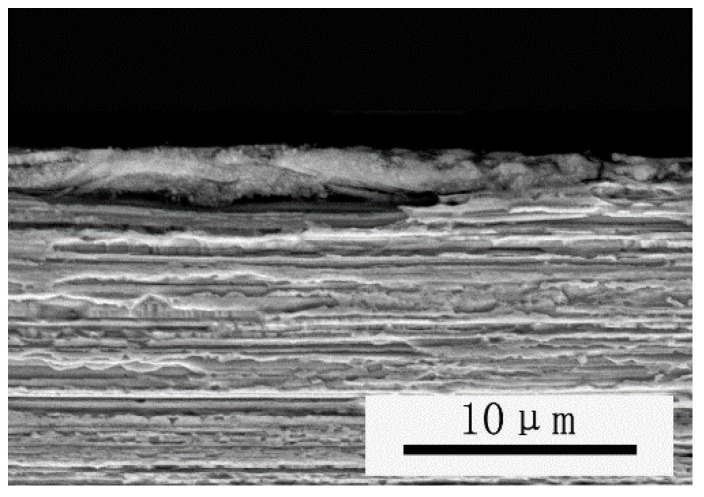
Edge section of wire material (typical regions).

Luke and his collaborators pointed out [20] that, in many cases, the fracture occurs mostly on the surface of the material (such as fatigue, corrosion, and wear). If large burrs or severe cracking defects occur on the surface of the tungsten wire during the tungsten wire production process, it is easy to result in wire breakage during further processing. In the process of being used as a microwave oven cathode, if there are surface defects such as burrs, abnormal ablation or electrode triggering failure, etc., will be caused. A comparison of the surface conditions of the wire prepared in Process 2 is shown in Figure 6. A comparison for the surface condition of the wire prepared in Process 2 is shown in Figure 6.

## 4. Experimental Analysis and Discussion

### 4.1. Analysis for Fiber-like Grains during Large Turntable Wire Drawing Processing of Tungsten Wires

Comprehensive analysis of the process factors that may affect the tungsten wires microstructure during the large turntable wire drawing processing: wire temperature, compression ratio, drawing speed, and mold temperature. In order to determine the factors that result in such a big change for the microstructure and properties of the φ0.50 mm tungsten wire, we made an analogy experiment among these factors. Based on the experimental results, we found that the heating temperature for the wire during processing is the main influencing factor affecting the size of the finished grains.

During the drawing process, temperature has a significant influence on the microstructure of the tungsten wires. As the tungsten wire can be processed only after the temperature reaching the plastic–brittle transition point [21], the tungsten wire processing process is a warm processing. In this experiment, the processing temperature of φ1.00 mm–0.50 mm tungsten wire is about 1050 °C–750 °C.

Zhao P proved that pure tungsten wire begins to recrystallize at 800 °C [22]. Additionally, for lanthanum-doped tungsten wires, as they are composites with rare earth oxides, which hinder the grain boundary motion, the recrystallization temperature should be increased accordingly [10,11,12]. However, the higher the heating temperature, the better the plasticity of tungsten and the higher the degree of dynamic recovery [18].

The inside of the φ1.00 mm tungsten wire has already formed its fibrous-like grains before the large turntable wire drawing processing starts. Therefore, the deformation of the microstructure during the large turntable wire drawing processing stage is realized by the dislocation motion and the coordinated deformation of the grain boundary motion [23]. The dislocation entanglement and dislocation cell structure appear after the plastic processing of the tungsten material [24]. The internal dislocation of the tungsten wires is more likely to slip, climb, and cross slip with a higher heating temperature [25]; this would help to eliminate the structural changes caused by plastic processing.

If the heating temperature reaches 0.3–0.5 Tm, a process called “dynamic polygonalization” will occur, which will lead to the widening of the internal structure of the tungsten wire. The drawing temperature has a great influence on the hardness of the wire. The heating temperature of process 1 and process 3 is 1050 °C, which is higher than 0.3 Tm (0.3 Tm 1023 °C), while the heating temperature of Process 2 and Process 4 is 950 °C, which is lower than 0.3 Tm. The microhardness of the tungsten wire produced by Processes 2 and 4 is higher than that of Process 1 and Process 3. The hardness and strength are positively correlated. Therefore, the hardness and strength of the material are higher at 950 °C.

### 4.2. Analysis of Surface Quality of Tungsten Wire

The key factors affecting the surface quality of the tungsten wire are the compression ratio and the difference between the mold temperature and the wire temperature (Δt).

**Figure 7:**
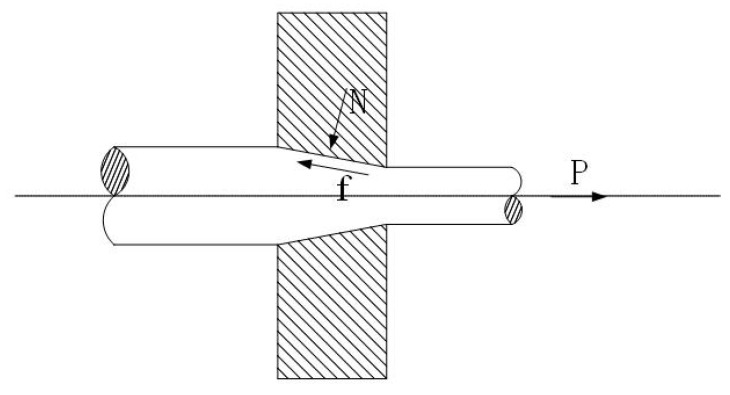
Schematic diagram of the force of the drawing process (N: pressure, P: drawing force, f: friction).

Shown in Figure 7, the compression ratio is high, and as the amount of compression for each die is relatively high, the pulling force and friction force are increased correspondingly; thus, problems such as surface friction and protrusion, etc., occur more frequently. The better the surface quality of the die hole, the lower the frictional force during the drawing of the tungsten wire, so the surface quality of the tungsten wire is better; the bigger the value of Δt, the greater the decrease in surface temperature when the tungsten wires pass through the die. Additionally, this would lead to a poor compatibility of deformation between the surface of the tungsten wires and the internal deformation. If the surface temperature is low, the deformation for the core becomes more difficult, and surface defects are more likely to occur.

Combined with the experimental results, it is considered that the optimum temperature for the further processing of lanthanum-doped tungsten wires with φ1 mm is 950 °C, and the temperature should be reduced by 25 °C for each subsequent processing.

### 4.3. Tungsten Wire Straightness Analysis

Straightness can reflect the performance of tungsten wires directly. The tungsten wire with poor straightness is prone to distortion and deformation during the process of magnetron cathode winding, and it leads to a negative impact on the subsequent processes of cathode preparation.

The main reason for the poor straightness of tungsten wires is that there are more residual internal stresses in the tungsten wires during the preparation process, and the uneven distribution of internal stress would result in curling and winding after the tungsten wire is prepared [13].

The key factors affecting the straightness of the tungsten wire are the compression ratio and Δt. During the preparation process, the tungsten wire is not stressed uniformly on the same section [26].

The compression ratio is an important factor affecting the straightness of the tungsten wire. When the compression ratio is too high, the residual stress distribution inside the tungsten wire is more uneven, and the drawing force and friction force increase with the increase in the compression ratio. The straightness of Process 2 and 3 is higher than that of Process 1 and 4, but the compression ratio should not be too small, otherwise the mold time and production cost of tungsten wire preparation will increase. Therefore, the preferred compression ratio is 15%.

The bigger the ΔT value, the greater the surface temperature drop as the tungsten wire passing through the mold. When the tungsten wire passes through the mold, the difference between the surface temperature and the internal temperature is larger, which tends to cause poor compatibility of deformation between the surface of the tungsten wire and the internal deformation. The surface temperature is low, the deformation is more difficult with respect to the core, and surface defects are likely to occur.

The force on the tungsten wires becomes more ununiform if the compression ratio is too big during the preparation process, and this would lead to the uneven distribution of internal stress. Moreover, the process with bigger compression ratio has a negative impact on the elimination of the residual internal stress inside the tungsten wires while preparing the wires with the same diameter, as the chance of dynamic recovery for the tungsten wire is less while the die times is less. If the value of Δt is bigger, the compatibility of deformation between the surface and the more uneven during the drawing process and this will lead to a poor straightness result for the tungsten wires.

## 5. Conclusions

(1) In the processing of φ1.00 mm–φ0.50 mm, the optimum preparation process is with a wire preparation temperature of 950–750 °C (the first die temperature is 950 °C, and the temperature is decreased about 25 °C each time for each die afterwards), a compression ratio of 15%, and a die temperature of 550–500 °C (the first die is 550 °C, and the subsequent die is gradually reduced). Because of the equipment limitations, the drawing speed is fixed at 0.19 m/s.
(2) The drawing temperature has a great influence on the hardness of the wire. The heating temperature of Process 1 and Process 3 is 1050 °C, which is higher than 0.3 Tm (0.3 Tm 1023 °C), while the heating temperature of Process 2 and Process 4 is 950 °C, which is lower than 0.3 Tm. The microhardness of tungsten wire produced by Processes 2 and 4 is higher than that of Process 1 and Process 3. The hardness and strength are positively correlated. Therefore, the hardness and strength of the material are higher at 950 °C.
(3) The compression ratio is an important factor affecting the straightness of the tungsten wire. When the compression ratio is too high, the residual stress distribution inside the tungsten wire is more uneven, and the drawing force and friction force increase with the increase in the compression ratio. The straightness of Process 2 and 3 is higher than that of Processes 1 and 4, but the compression ratio should not be too small, otherwise the mold time and production cost of tungsten wire preparation will increase. Therefore, the preferred compression ratio is 15%.
(4) The average grain size of the four processes is 0.1684 μm, 0.0982 μm, 0.1361 μm, and 0.1024 μm. It can be seen that the sizes of grains prepared by Process 2 are finer. In addition, the surface quality of the wire obtained by this process is excellent, which is suitable for microwave oven applications and can effectively improve the reliability of cathode work.

## Figures and Tables

**Table 1:** Process parameter table.

	Compression Ratio	Mold Temperature (°C)	Wire Heating Temperature (about 25 °C Decrease per Die)
Process 1	22%	550	1050
Process 2	15%	550	950
Process 3	15%	550	1050
Process 4	22%	550	950

**Table 2:** Different types of tungsten wire straightness.

	Process 1	Process 2	Process 3	Process 4
Straightness	32%	90%	34%	52%
